# Capivasertib-Induced Diabetic Ketoacidosis in a Patient With Estrogen Receptor-Positive/Human Epidermal Growth Factor Receptor 2-Negative (ER+/HER2-) Metastatic Breast Cancer and No Prior History of Diabetes Mellitus: A Case Report

**DOI:** 10.7759/cureus.63710

**Published:** 2024-07-02

**Authors:** Yoan E Rodriguez, Rishu Batra, Kunal Patel, Suzanne Martinez

**Affiliations:** 1 Internal Medicine, HCA Florida Orange Park Hospital, Orange Park, USA; 2 Internal Medicine/Endocrinology, HCA Florida Orange Park Hospital, Orange Park, USA

**Keywords:** er+/her2- metastatic breast cancer, akt inhibitor's adverse effect, diabetic ketoacidosis (dka), breast cancer, capivasertib

## Abstract

Capivasertib, a pan-AKT inhibitor, has shown promising efficacy in treating metastatic tumors harboring the *AKT1 E17K* mutation. However, its use is associated with notable adverse events, including hyperglycemia, which may impact treatment outcomes. This case describes a patient with estrogen receptor-positive/human epidermal growth factor receptor 2-negative (ER+/HER2-) metastatic breast cancer and no prior history of diabetes who developed diabetic ketoacidosis (DKA) following capivasertib therapy.

## Introduction

Capivasertib, an oral selective inhibitor of the serine/threonine kinase AKT, has garnered significant attention in treating various cancers, particularly those with aberrations in the PI3K/AKT signaling pathway. The phosphoinositide 3-kinase (PI3K) pathway is crucial in cell physiology as it carries out the transmission of various extracellular stimuli through a signaling cascade. AKT is a downstream target of this pathway, which is crucial for the survival, proliferation, and glucose metabolism of cells [1]. Therefore, several studies have documented the efficacy of capivasertib in managing hormone receptor-positive breast cancer and tumors harboring *AKT1* mutations, highlighting its potential as a pivotal component in targeted cancer therapy.

Kalinsky et al. (2021) demonstrated the promising activity of capivasertib in patients with *AKT1 E17K*-mutated tumors, indicating a substantial therapeutic benefit [2]. Similarly, the phase 2 trial by Jones et al. (2020) provided evidence of improved progression-free survival with the combination of fulvestrant and capivasertib in metastatic estrogen receptor-positive breast cancer, compared to placebo [3]. Hyman et al. (2017) further corroborated the efficacy of AKT inhibition in solid tumors with *AKT1* mutations, reinforcing the rationale for utilizing capivasertib in specific oncogenic contexts [4].

Moreover, a comprehensive review by Andrikopoulou et al. (2022) elucidated the emerging role of capivasertib in breast cancer, encompassing various clinical trials and potential applications [5]. The recent study by Turner et al. (2023) on hormone receptor-positive advanced breast cancer showcased significant improvements in progression-free and overall survival with capivasertib, cementing its status as a potent therapeutic agent [6].

Despite these promising outcomes, the clinical use of capivasertib is not without challenges. One notable adverse effect is hyperglycemia, a condition that can complicate treatment and impact patient quality of life [2,3]. Hyperglycemia induced by capivasertib is attributed to its inhibition of the AKT pathway, a critical regulator of glucose metabolism [5]. This case report aims to explore the occurrence of hyperglycemia in a patient undergoing capivasertib treatment, providing insights into the management and implications of this side effect.

## Case presentation

**Figure 1:**
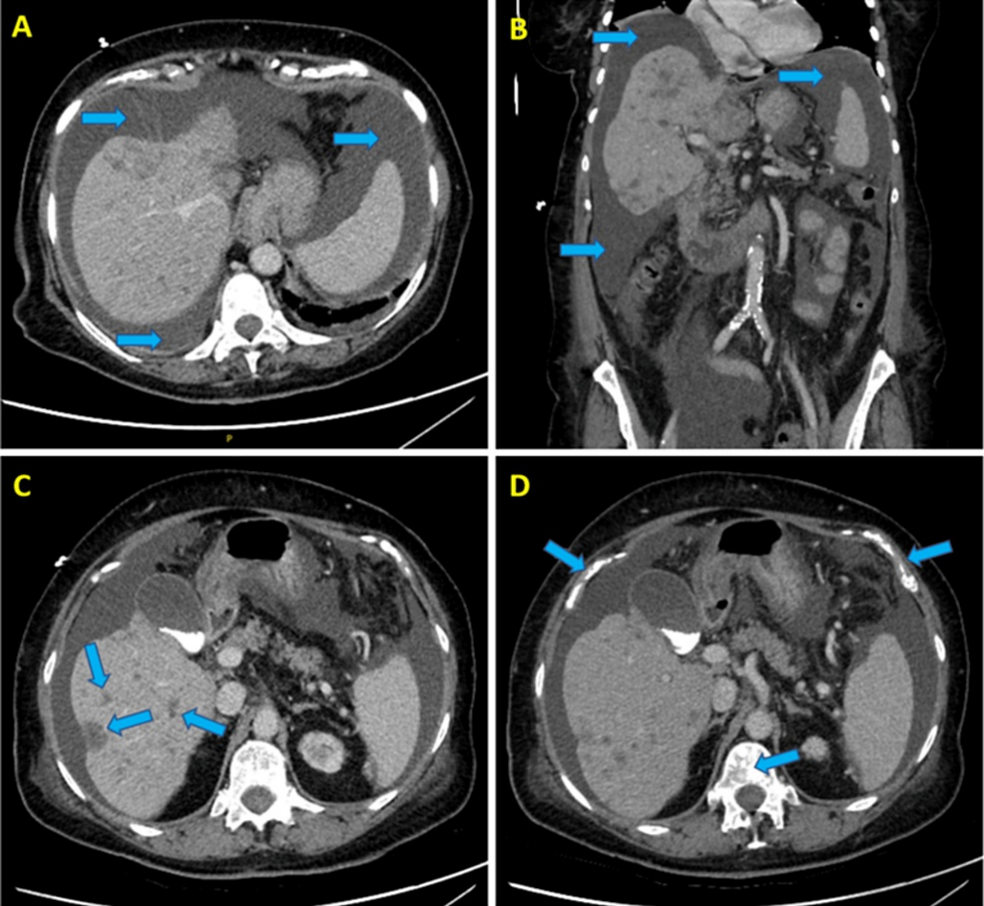
Computed tomography (CT) scan of the abdomen and pelvis with intravenous contrast. (A) The axial view and (B) coronal view show a moderate-to-large amount of free fluid within the abdominal (peritoneal) cavity, indicated by blue arrows. (C) In the axial view, multiple hepatic lesions are marked by blue arrows, which are likely indicative of metastases. (D) The axial view reveals several areas of increased bone density (sclerosis) throughout the skeleton, highlighted by blue arrows, suggesting the presence of metastatic disease.

**Figure 2:**
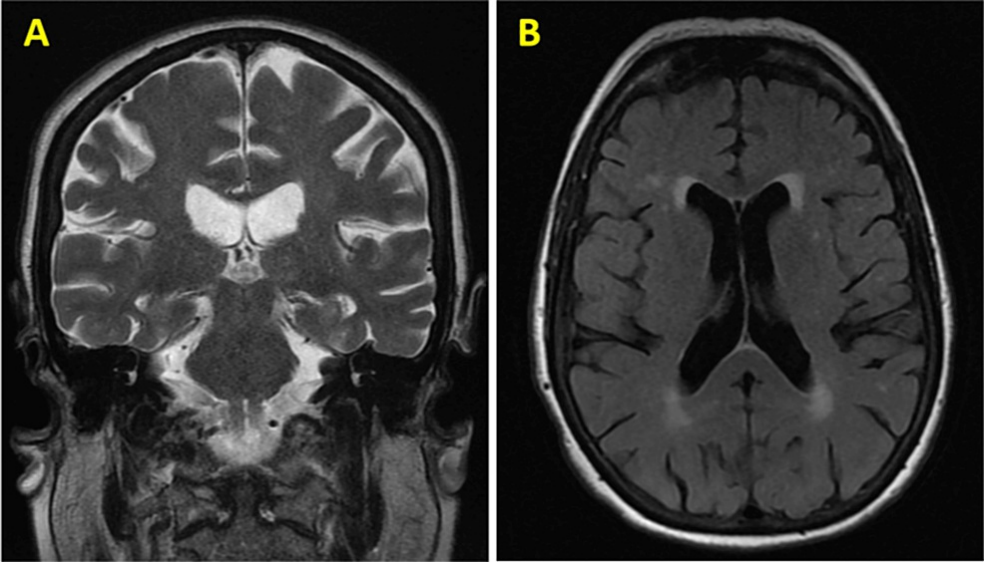
Magnetic resonance imaging (MRI) of the brain without intravenous contrast. (A) The coronal view and (B) axial view of the MRI brain reveal no signs of acute intracranial pathology. This includes the absence of acute infarct, mass effect, hydrocephalus, midline shift, or fluid collection.

A 77-year-old female with a past medical history of chronic kidney disease stage 3b and stage IV estrogen receptor-positive (ER+) and human epidermal growth factor receptor 2-negative (HER2-) breast cancer with metastasis to the bone and liver diagnosed three years before presented with altered mental status for one day. The patient also complained of polydipsia and decreased urine output. The history obtained by the daughter described her as confused and not at her baseline. The patient previously received taxanes, capecitabine, and doxorubicin due to breast cancer progression while receiving hormonal therapy plus a cyclin-dependent kinase 4 and 6 (CDK4/6) inhibitor. The patient’s condition progressed again around two months before the current admission, when she presented with bloating and generalized weakness. A computed tomography (CT) scan of the abdomen and pelvis revealed ascites plus multiple hepatic and sclerotic bony lesions consistent with metastatic disease, as shown in Figure 1. Additionally, the brain MRI result was negative for brain metastases or any other acute intracranial abnormality at that time, as shown in Figure 2.

After hospital discharge, the patient started salvage therapy with capvasertib and fulvestrant 10 days prior to the onset of altered mental status. On presentation, the patient was noted to be confused. The patient’s vital signs were significant for hypotension, with a blood pressure of 66/37 mmHg and a respiratory rate of 27 breaths per minute, but all other vital signs were within normal limits. The physical exam showed dry mucous membranes and fine rales at the lung bases but was otherwise unremarkable. The laboratory examinations showed an arterial blood gas with a pH of 7.3, decreased bicarbonate (HCO_3_) of 8.4, and decreased partial pressure of carbon dioxide (pCO_2_) of 16.4, indicating severe metabolic acidosis compensated by respiratory alkalosis.

The blood tests revealed hyperglycemia with a blood glucose of 718 mg/dL, elevated beta-hydroxybutyrate of 5.5 mmol/L, increased anion gap of 26.4 mmol/L, decreased bicarbonate level of 12.6 mEq/L, and lactic acidosis of 5.2 mmol/L consistent with high anion gap metabolic acidosis. However, the patient had no prior diagnosis of diabetes and reported no history of hyperglycemia. Additionally, her glycated hemoglobin (HbA1c) was within the normal range at 5.0 during admission. A urinalysis revealed elevated ketones, positive leukocyte esterase, elevated urine white blood cells, positive glucose, and many bacteria and yeast consistent with a urinary tract infection (UTI). She also had an acute renal injury with a creatinine of 5.12 mg/dL with a prior baseline creatinine of 1.3-1.6 mg/dL.

**Figure 3:**
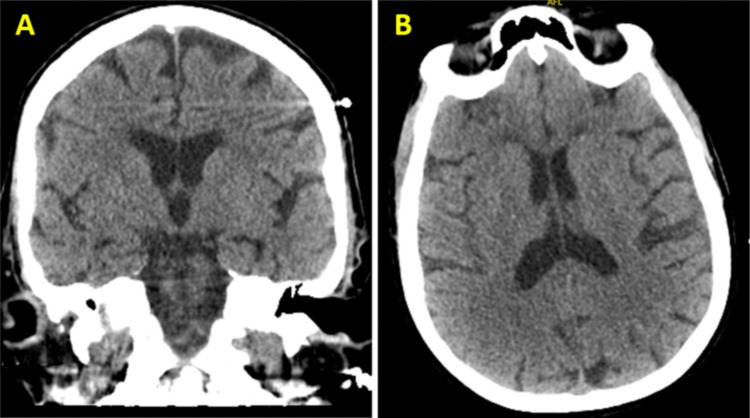
Computed tomography (CT) scan of the brain without intravenous contrast. (A) The coronal and (B) axial brain scans reveal no signs of acute intracranial pathology. This includes a normal density of the brain parenchyma, the absence of acute intracranial hemorrhage, and no indications of abnormal masses, growths, mass effect, midline shift, or hydrocephalus.

**Figure 4:**
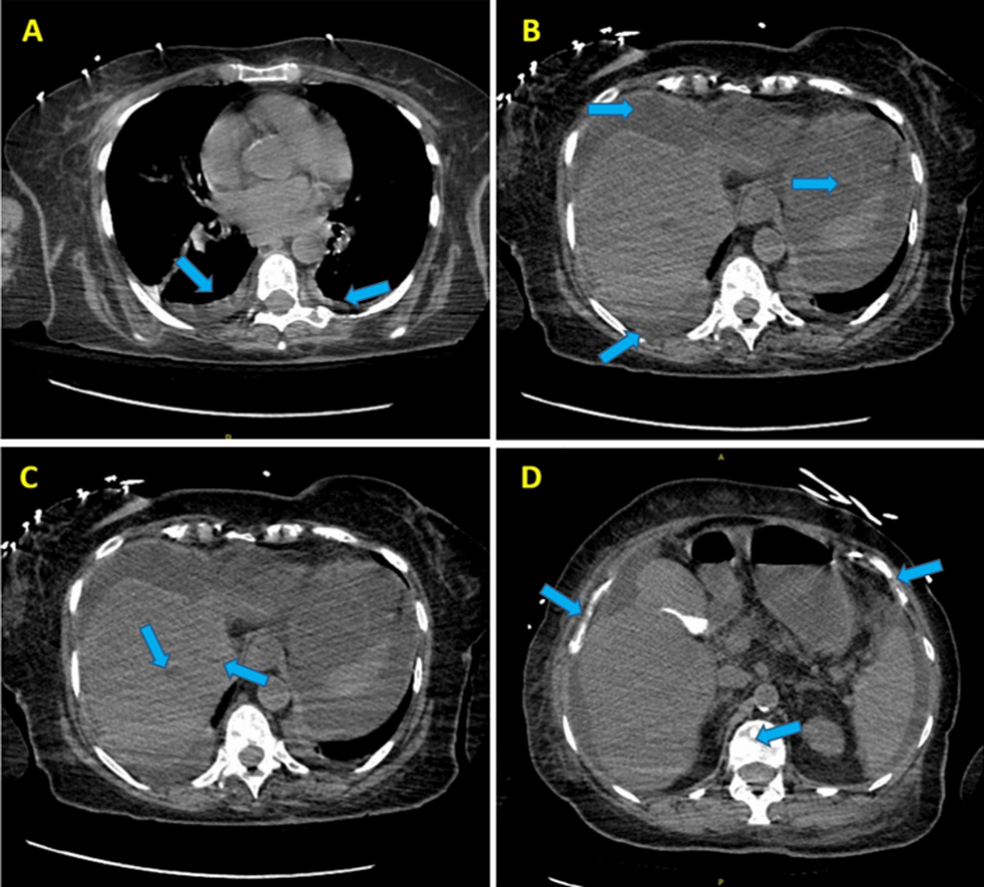
Computed tomography (CT) scan of the abdomen and pelvis without intravenous contrast. (A) The axial view shows small fluid collections around the lungs, likely indicating bilateral pleural effusions at the lung bases, marked by blue arrows. (B) The axial view reveals a moderate-to-large amount of free fluid in the abdomen (peritoneal cavity), highlighted by blue arrows. (C) The axial view displays multiple hepatic lesions pointed out by blue arrows, likely indicative of metastatic disease, but their characterization is uncertain without contrast, unlike the previous contrast-enhanced exam. (D) The axial view presents multiple areas of increased bone density (sclerosis) throughout the skeleton, identified by blue arrows, suggesting metastatic disease. The scan was conducted without intravenous contrast due to the patient's renal function. While this scan supplies valuable information, contrast enhancement could provide a more precise assessment of blood flow and help identify lesions, such as iso-dense lesions, which may be challenging to detect on a non-contrast scan.

The CT scan of the head was negative for any acute intracranial abnormality (Figure 3). The CT scan of the abdomen and pelvis revealed bilateral pleural effusions, ascites, and hepatic and bony metastatic lesions, which were unchanged from prior imaging, as shown in Figure 4. The high anion gap metabolic acidosis, ketosis, and hyperglycemia were consistent with diabetic ketoacidosis (DKA). Given the normal HbA1c level on admission and symptom onset occurring soon after starting capivasertib therapy, for which hyperglycemia and insulin resistance are known adverse effects, a diagnosis of capivasertib-induced DKA was given.

**Figure 5:**
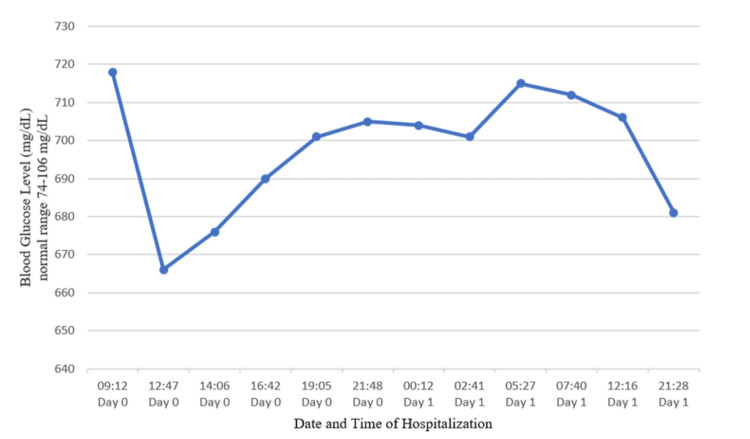
Blood glucose trend. This graph indicates that the patient's blood glucose levels are persistently in the range of 600-700 mg/dL, even while receiving an insulin infusion.

Intravenous fluids and insulin therapy (insulin drip at 0.1 U/kg/hour rate and titrated following the DKA protocol of our institution) were initiated to address hyperglycemia. To address any potential underlying infection, broad-spectrum antibiotics with vancomycin and cefepime were administered. Urine cultures were sent to identify the causative organism. Vasopressors were initiated for the patient, who was then admitted to the intensive care unit for the management of septic shock, acute renal failure, acute metabolic encephalopathy, and DKA. The patient's hypotension resolved, and mental status normalized within 24 hours. However, the patient had persistent hyperglycemia over the next 48 hours, with blood glucose levels remaining stubbornly in the 600-700 mg/dL range, as shown in Figure 5, despite the patient receiving large amounts of insulin with an insulin drip. Blood cultures were negative for any microorganism growth. However, a urine culture grew *Candida albicans*, indicating a fungal UTI. Antifungal medications were not added to the treatment regimen due to patient and family refusal.

The patient's clinical course was further complicated on day one of admission by worsening renal failure with no urine output. Despite medical recommendations, the patient and family declined hemodialysis. Additionally, the patient and family opted against further invasive interventions, such as intravenous antibiotics, mechanical ventilation, or vasopressor therapy. The patient's decision regarding hemodialysis and further invasive interventions was respected, and supportive care measures were provided to optimize her comfort and quality of life. Ultimately, the patient and her family elected to pursue comfort care, and she was discharged to hospice.

## Discussion

Endocrine therapy, usually with an aromatase inhibitor in post-menopausal females, combined with a CDK4/6 inhibitor is the first-line treatment for advanced breast cancer positive for estrogen and/or progesterone (ER+/PR+) hormone receptor and HER2- overexpression [6]. Nonetheless, disease progression is common among these patients, leading to a clinical challenge in their treatment. Capivasertib-fulvestrant therapy has shown significantly longer progression-free survival among these patients. On the other hand, treating patients with PI3K/AKT pathway inhibitors presents a significant challenge due to their associated toxicities, which include stomatitis, rash, hyperglycemia, nausea, diarrhea, and fatigue [7]. This case highlights the potential for capivasertib to induce new-onset diabetes mellitus (de novo diabetes) and resultant DKA, even in patients without a pre-existing diabetic condition.

Hyperglycemia occurs in up to 80% of subjects on clinical trials for PI3K/AKT inhibitors since this pathway controls insulin sensitivity and glucose metabolism [8]. The inhibition of AKT activity can block adipocyte insulin-dependent glucose uptake and regulation. Additionally, the genes encoding most glycolytic enzymes are under dominant transcriptional control by the PI3K/AKT pathway; for example, glycogen synthase kinase-3 is a substrate of AKT, which regulates hepatic glycogenolysis and glucose uptake [1,8]. Consequently, the occurrence of hyperglycemia is an on-target effect of ATK inhibitors that requires an effective clinical approach to proactively address and mitigate this response. Additionally, the superimposed acute illness (UTI and septic shock) may have further contributed to the metabolic derangement in this case. The initial presentation of confusion and altered mental status is a crucial sign that should not be overlooked in DKA. The persistent hyperglycemia despite high insulin doses suggests a significant impairment in insulin sensitivity, possibly caused by capivasertib.

There are no clear guidelines available for managing AKT inhibitor-induced hyperglycemia. Prior to initiating treatment, it is advisable to start monitoring glucose levels at home and implementing more intensive dietary modifications. Metformin is recommended as the first-line therapy for AKT inhibitor-induced hyperglycemia in some clinical trials [8]. If hyperglycemia persists despite the use of metformin, it may be appropriate to incorporate sodium-glucose cotransporter-2 (SGLT2) inhibitors into the treatment regimen, or other potentially effective pharmacologic options such as sulfonylureas or insulin should be considered [8]. Endocrinology consultation is also highly recommended in this setting [1]. Additionally, regular blood glucose monitoring is essential during capivasertib therapy. Dose reduction or discontinuation of capivasertib may be necessary, depending on the severity of hyperglycemia.

## Conclusions

Clinicians prescribing capivasertib should be aware of the potential for DKA, even in patients without a history of diabetes. Regular blood sugar monitoring is essential during capivasertib therapy. At the first sign of hyperglycemia, a dose reduction or discontinuation of capivasertib may be necessary. Capivasertib has the potential to induce severe insulin resistance, requiring aggressive insulin management. However, this is a single-case report, and further research is needed to determine the overall risk of insulin resistance and DKA associated with capivasertib. As more AKT inhibitors are receiving approval or entering clinical trials, there is a need to further investigate AKT inhibition-induced hyperglycemia and establish effective management strategies.
